# Reference Gene Selection for RT-qPCR Analysis in Maize Kernels Inoculated with *Aspergillus flavus*

**DOI:** 10.3390/toxins13060386

**Published:** 2021-05-28

**Authors:** Dafne Alves Oliveira, Juliet D. Tang, Marilyn L. Warburton

**Affiliations:** 1 Department of Biochemistry, Molecular Biology, Entomology and Plant Pathology, Mississippi State University, Starkville, MS 39762, USA; 2 USDA FS, Forest Products Laboratory, Starkville, MS 39759, USA; juliet.d.tang@usda.gov; 3 USDA ARS Corn Host Plant Resistance Research Unit, Starkville, MS 39762, USA; marilyn.warburton@usda.gov

**Keywords:** candidate gene, gene expression, aflatoxin

## Abstract

Resistance against infection by the fungus *Aspergillus flavus* Link in commercial maize (*Zea mays* L.) is the topic of many studies, but few studies have investigated the effects of *A. flavus* infection on gene expression levels in ear kernels. A crucial component of gene expression profiling by RT-qPCR is having a reliable set of reference genes that show relatively constant expression across the treatments and phenotypes under study. Currently, however, there is no published information on reference genes suitable for measuring changes in kernel gene expression levels after infection with *A. flavus*. Thus, in this study, six candidate reference genes (*ACT1*, *β-Tub2*, *eIF4A2*, *TATA*, *EFIα*, and *GAPDH*) were evaluated and ranked according to their expression stability. The genes were amplified from first-strand cDNA samples synthesized from kernels of two susceptible and two resistant maize lines that were either inoculated with *A. flavus* or water or not inoculated. Three software packages were used to calculate and rank the stability of expression for these genesgeNorm, NormFinder, and BestKeeper. The analysis revealed that the most stable genes to normalize expression levels from maize kernels responding to *A. flavus* inoculation and wounding were *ACT1*, *EFIα*, and *eIF4A2.*

## 1. Introduction

Many commercial maize (*Zea mays* L.) varieties are highly susceptible to fungal pathogens, including *Aspergillus flavus* Link. This fungus produces aflatoxin, the accumulation of which causes critical health and economic problems [1]. Over 200 genes have been proposed in the current literature as candidates that may help the maize plant suppress the effects of *A. flavus* [2] or the production of aflatoxin. However, the role of the majority of these genes in resistance still needs to be validated. One accurate method to perform such validation is by verifying gene expression changes following fungal infection via RT-qPCR (reverse transcription quantitative PCR) [3].

One of the preferred methods to quantify gene expression patterns via RT-qPCR is the 2^−ΔΔCq^ method proposed by Livak and Schmittgen [4]. This method measures the relative change in target transcripts between a treated and an untreated control sample, and it relies heavily on the normalization of the acquired Cq (quantitation cycle) values of all samples. Without the adjustment of the variation in the reverse-transcription yields and efficiency of amplification of mRNA, the comparison across different samples is meaningless [5]. This normalization is usually done using a baseline reference gene, which is stably expressed across all samples and treatments evaluated in the study. Thus, the selection of such a reference gene is vital to achieving reliable results, because if the reference gene that was chosen is regulated (and changed) by experimental conditions, this will lead to uninterpretable results [6]. Although the correct selection and use of stably expressed reference genes are part of the MIQE (minimum information for publication of qPCR experiments) guidelines of expression studies [7], a surprising number of published gene expression data using RT-qPCR still rely on the use of a single unvalidated reference gene [8].

Although studies to select reference genes suitable for RT-qPCR in maize under different stress conditions have been published in the literature [9,10,11], there is no information on validated reference genes to perform quality gene expression analysis on maize kernels under fungal attack (particularly *A. flavus*). This study attempts to fill this gap by evaluating the expression stability of six candidate reference genes in maize kernels, namely, *ACT1*, *β-Tub2*, *eIF4A2*, *TATA*, *EFIα*, and *GAPDH*. These genes are responsible for basic cell functions in eukaryotic cells, and, as such, expected to be constitutively expressed. They were tested for stability of gene expression in maize kernels from two aflatoxin accumulation resistant (Mp313E and Mp719) and two susceptible (Va35 and B73) maize lines in response to the inoculation of *A. flavus* spores or double distilled autoclaved water. The most stable reference genes identified will allow the normalization of genes studied by RT-qPCR to determine the change in expression following fungal inoculation.

## 2. Results

Analysis of the melt curve for each reference gene showed the specificity of the amplification product (Supplementary Figure S1). The efficiencies of amplification of the selected primers ranged between 92% to 108% (R^2^), where the *TATA* gene had the lowest efficiency, and the *EFIα* gene had the highest (Table 1). The *β-tub2* gene failed to amplify at the most diluted point of the curve, thus producing a standard curve of only four points instead of five; all other genes had five (Supplementary Figure S2).

**Figure 1:**
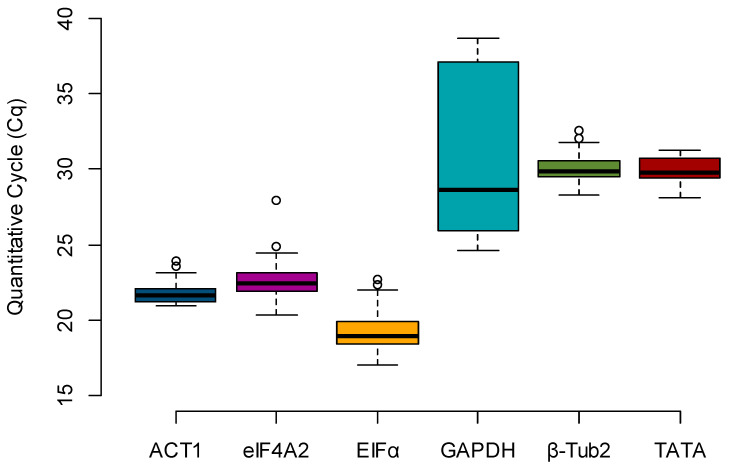
The expression profile for candidate reference genes over biological replications and treatments (*n* = 36). The length of the box represents the distance between the 25th (bottom) and 75th (top) percentiles, and the difference between these is proportional to the stability index. The horizontal line in the box interior represents the group median, and the whiskers are extended to the group’s minimum (bottom) and maximum (top) values, not including outliers of the data, which are represented as white circles.

Box plots were created to evaluate the expression profile of the candidate reference genes, using the raw Cq values across all samples (*n* = 36) (Figure 1). The most highly expressed gene, e.g., the one with the lowest Cq, was *EFIα* (mean Cq = 19.315), and the least expressed gene was *GAPDH* (mean Cq = 31.324). By calculating the difference between the upper (75th) and lower (25th) percentile (ΔP), it is possible to determine the stability index, which is proportional to the spread of the data [12]. Considering only the Cq value spread (ΔP), the stability rank was *ACT1* (0.955), *β-Tub2* (0.995), *eIF4A2* (1.187), *TATA* (1.277), *EFIα* (1.494), and *GAPDH* (11.152) (Table 2).

**Figure 2:**
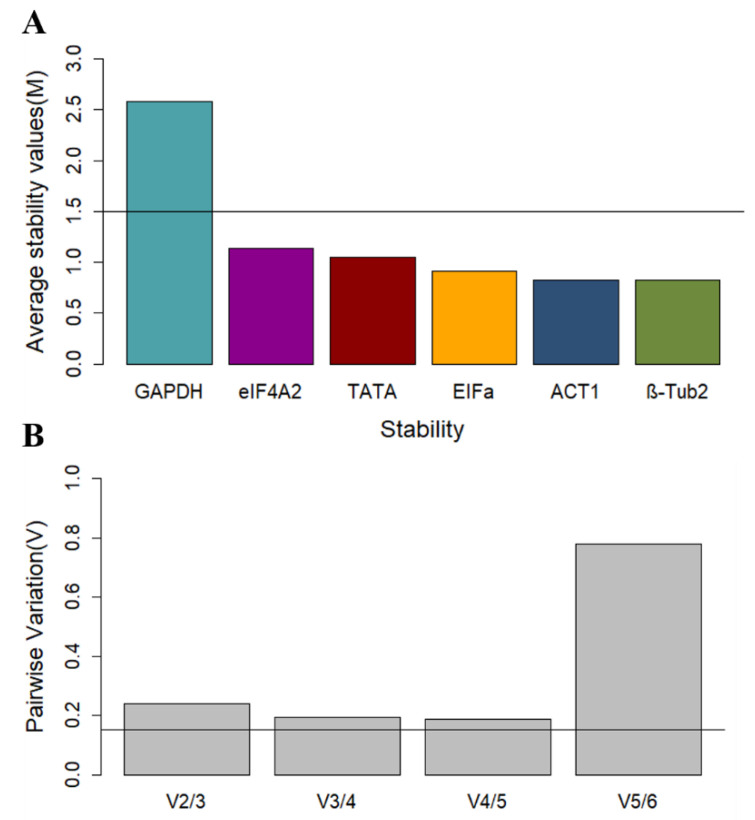
(**A**) Mean of stability value (M) for each candidate reference gene calculated by the geNorm algorithm over biological replications and treatments. Genes are ranked from the least (left) to the most (right) stable. Only genes with an M < 1.5 are considered useful with stable expression. (**B**) Average pairwise variation (V) calculated by the geNorm algorithm to determine the optimal number of reference genes for normalization analysis. Pairwise values that are below the 0.15 threshold indicate that the addition of another reference gene will have no significant improvement on normalization analysis.

To calculate the stability of reference genes using geNorm [13], the Cq raw data must first be converted to relative expression levels (ΔCq). This normalized data is then used to calculate the expression stability value (M), which has an ideal threshold of 1.5. The stability of the genes is inversely proportional to M, thus the lower this value, the more stable the gene. *GAPDH* was above the M threshold value, and the three most stable genes were *ACT1*, *EFIα*, and *eIF4A2* (Figure 2A).

Another feature of the geNorm algorithm is to calculate the optimal number of reference genes needed for accurate normalization. Following this, pairwise variation values (V) are calculated, and values below 0.15 (Vn/Vn+1 < 0.15) indicate that the “N+1” number of genes is not necessary, since an addition will not significantly improve the accuracy of normalization [13]. The geNorm manual recommends the removal of the least stable genes only up to the point that the optimal number of genes remain so that the algorithm can calculate an accurate M value using the data from the most stable genes only. The software performs this step progressively, eliminating one gene at a time until only the optimal number of genes is left, then ranking the genes for stability. The results of the pairwise variation analysis are presented for the genes in this study in Figure 2, which suggests that the three least stable genes can be eliminated from the analysis set. Based on the calculated M values, geNorm ranks the remaining three most stable genes (in order of most to least stable) as *β-Tub2* > *ACT1* > *EFIαI* (Table 3).

The NormFinder [14] software package, like geNorm, calculates gene stability using relative expression levels (ΔCq); thus, the data used for calculation in geNorm were also used in NormFinder. Unlike geNorm, NormFinder allows the user to group the data according to categorical variables. With NormFinder, the gene *GAPDH* ranked as the least stable, and the genes *EFIα* and *eIF4A2* were the most stable (Table 3).

BestKeeper calculates the stability of the reference genes based on the standard deviation of the raw Cq values [15]. Thus, genes with a higher standard deviation, e.g., with broader dispersion of the data points, are less stable than genes with a lower standard deviation. With this stability measurement, the gene *ACT1* was the topmost stable candidate, followed by *β-Tub2* and *TATA*, which were tied for second; *GAPDH* was the least stable by a considerable margin (Table 3).

The gene *β-Tub2* was among the top three most stable candidates for all the algorithms used. However, it is essential to note that this gene had a low expression in all maize kernels in this study, as the detected Cq averaged only 30.062 and the lowest Cq was 28.307 (Figure 1). A highly expressed reference gene is important in gene expression analysis that uses the 2^−ΔΔCq^ method [4] since a low expression reference gene could result in an unrealistic fold change. A similarly low expression can be observed for the gene *TATA*, which ranked as the third most stable gene when using BestKeeper (Table 3). Thus, the three best reference genes for expression and stability were *ACT1*, *EFIα*, and *eIF4A2*, and the least stable was the gene *GAPDH*.

**Figure 3:**
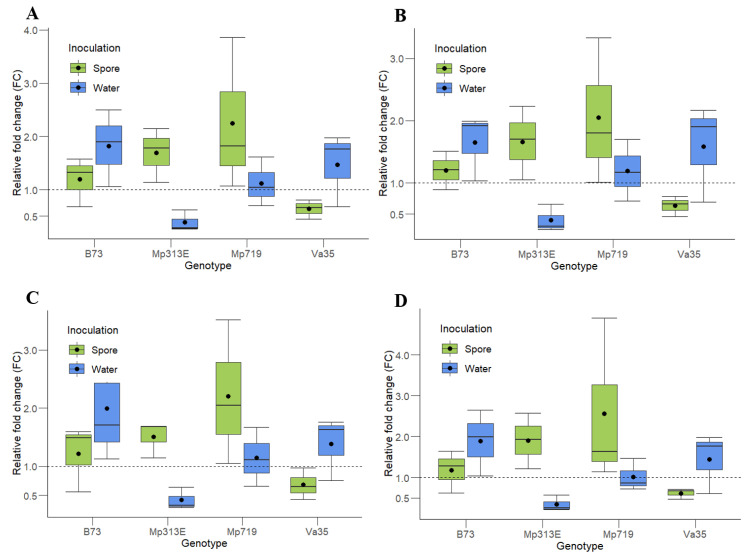
Expression analysis of the gene *Zm00001d020612* in maize kernels normalized with (**A**) *ACT1*, *EFIα*, and *eIF4A2*, (**B**) *ACT1* and *EFIα*, (**C**) *ACT1* and *eIF4A2*, and (**D**) *EFIα*, and *eIF4A2* as the reference genes. The control treatment used to calculate the relative expression of the samples inoculated with *A. flavus* spores are samples inoculated with water, and for samples inoculated with water, the control treatments used were uninoculated samples of the same genotype. Genes in which FC < 1 was downregulated in response to the stress applied and genes in which the mean FC > 1 was upregulated. The length of the box represents the distance between the 25th (bottom) and 75th (top) percentiles; the horizontal line in the box interior represents the group median; the black dot inside the boxes represents the group mean; and the whiskers are extended to the group’s minimum (bottom) and maximum (top) values, not including outliers of the data.

To evaluate the reliability of the candidate reference genes, expression analysis of the maize gene *Zm00001d020612* was performed (Figure 3). The product of this gene is a choline/ethanolamine kinase responsible for catalyzing the first step in the biosynthesis of phospholipids, which are essential for the formation of cell membranes [16] and the first place of contact and recognition for plant pathogens. This gene has shown a significant increase in RNA transcript in maize leaves 72 h post-inoculation of the *Colletotrichum graminicola* fungus [17]. Additionally, this gene was identified as one of the top candidates to reduce aflatoxin accumulation in a GWAS (Genome-Wide Association Study) analysis performed in maize challenged with *A. flavus* [18].

Since the pairwise analysis from geNorm suggested that a minimum of three reference genes be used to normalize the data, the gene of interest (*Zm00001d020612*) from each maize was normalized using the three most stable and highly expressed genes, *ACT1*, *EFIα*, and *eIF4A2*, together or in combinations of two (*ACT1* and *EFIα*; *ACT1* and *eIF4A2*; and *EFIα* and *eIF4A2*) *(*Figure 3). The normalization was performed using the method proposed by Livak and Schmittgen [4].

The same pattern of relative fold change (FC) is observed in all the genotypes and treatments using all of the two- and three-gene combinations of reference genes. The resistant maize genotypes, MP313E and Mp719, showed an approximately two-fold upregulation when inoculated with *A. flavus* spores, while the susceptible lines, Va35 and B73, show no differences in the gene expression (FC = 1) for the same treatment. In response to the water inoculation, no maize line except for Mp313E showed any difference in expression of the gene *Zm00001d020612*.

To confirm the unsuitability of the gene *GAPDH* as a reference gene, the same maize samples were analyzed for the expression of the gene *Zm00001d020612* following the same parameters described on Figure 3. The Cq values for *GAPDH* were highly variable among the samples, ranging from 24.629 to 38.629 (Figure 1), which transposed to the normalization of the evaluated samples. The most affected genotype was the susceptible line Va35, which showed an FC ranging from 0.5 to 45 for the water inoculation treatment (data not shown) versus 0.5- to 2-fold when the same samples were normalized by the most stable reference genes (Figure 3). The resistant genotype Mp313E showed an unrealistic downregulation of *Zm00001d020612* in response to the inoculation of *A. flavus* instead of an upregulation, as seen with the other reference genes. The high variance around the expression profiles normalized with *GAPDH* makes comparisons between treatments or genotypes uninformative.

## 3. Discussion

The current literature is populated with over 200 candidate genes that may play an essential role in the interaction between maize and *A. flavus* [2]. Before these genes can become useful in maize breeding programs designed for *A. flavus* resistance, they must first be validated [19]. To achieve the objective of this study and identify reference genes for candidate gene validation using RT-qPCR, three different software packages (geNorm, NormFinder, and BestKeeper) were used to rank the stability of the selected candidate reference genes. When comparing the ranking set by each software package, the three most stable genes varied among the different analysis sets (Table 3). By eliminating the three genes that had expression values too low or too variable to be useful (*β-Tub2*, *TATA*, and *GAPDH*), we found the remaining three genes to be the most stable and suitable for normalization, in the order *ACT1*, *eIF4A2*, and *EFIα*. As expected from their predicted housekeeping functions (Table 1), these three genes had low Cq values, indicating high expression in maize kernels (Figure 1).

The gene *ACT1* encodes actin, of which the cytoskeleton of plant cells is composed, and which plays an important role in intracellular movements [20]. The two other genes, *eIF4A2* and *EFIα*, participate in mRNA translation, a step in the expression of every gene that occurs continuously in all living cells. The gene *eIF4A2* encodes a subunit of the *eIF4F* initiation factor complex that participates in the first step of the translation process [21]. This protein is a DEAD-box helicase that mediates recognition of the 5′-cap structure and unwinds the mRNA so that other eIFs can interact with the small subunit of the ribosome and initiate the translation process. The *EFIα* gene, the most highly expressed gene in this study, encodes the eukaryotic translation elongation factor 1A, which is the most abundant synthesis factor protein in a cell [22]. This GTP-binding protein binds the aminoacetyl-tRNAs to ribosomes during translation and releases them when the correct codon anticodon match is made. *EFIα* also participates in other cell functions, including nuclear export of tRNAs, protein degradation, apoptosis, and viral propagation [23].

This study has identified three reference genes suitable for normalization of expression data from maize kernels responding to *A. flavus* contamination and to wounding, which may be caused by fungal infection or by insect feeding. It can be predicted that in a study with similar characteristics to this one, these same three genes will maintain their stability, as shown in our results. Studies that identify possible candidate genes for resistance to biotic stresses, such as fungal pathogen and insect resistance, have multiplied with the publication of genetic, proteomic, and metabolomic studies. As a first step in validating these biotic stress resistance candidates, RT-qPCR, any of these three stable reference genes (and preferably two) can be used to create gene expression profiles for normalization of candidate genes. It should be noted, however, that the selection of reference genes for normalization of gene expression studies must be customized to the specific study, as different treatments might influence all but the most basic cell activities.

## 4. Materials and Methods

### 4.1. Plant Tissue and Treatments

The maize germplasm chosen for this research included two aflatoxin accumulation resistant lines (Mp313E and Mp719) and two susceptible lines (Va35 and B73). Each line was grown in a randomized complete block design with plant replicates in the experimental field at the Mississippi State University R. R. Foil Plant Science Research Center. Eighteen days after mid-silk (when half of the primary ears showed silk), the kernels were at the milk stage (R3) [24] and were inoculated with fungal spores. From each genotype, three ears were randomly selected to receive one of the following treatments inoculation of spores from the toxigenic *A. flavus* strain NRRL 3357; water-inoculation containing double distilled autoclaved water; or no inoculation.

The *A. flavus* inoculum for each strain was prepared by growing the strain on sterile corn cob grits (size 2040, Grit-O-Cobs, Maumee, Ohio) in 500 mL flasks, containing 50 g of grits and 100 mL sterile distilled water and incubated at 28 °C for 21 days. Conidia were obtained by washing the grits with a mixture of 500 mL of sterile distilled water and 0.1% Tween 20 (to prevent conidial clumping). The liquid containing the spores was then filtered through four layers of cheesecloth. Conidial concentration was calculated using a hemocytometer, and the final inoculum concentration was diluted to 9 × 10^7^ conidia/mL using sterile distilled water.

Inoculation of corn ears was performed by peeling back the husk and injecting the maize kernels with the eye of a size 12 quilting needle (Entaco Limited, Worcestershire, England, Cat. No. JJ12012) extending 2 mm from the base of a pencil eraser. The needle eye was dipped in the appropriate inoculum (conidia or water) before inoculating the maize kernels. The kernels were inoculated in two rows, alternating with uninoculated rows between the inoculated rows. After inoculation, the husk was put back around the ear, which was covered with two paper shoot bags and secured in position with rubber bands. Maize ears were collected three days after inoculation (DAI); the inoculated kernels were removed from the ears and flash-frozen in liquid nitrogen and immediately stored at −80 °C until RNA extraction.

### 4.2. Total RNA Extraction and cDNA Synthesis

Total RNA was extracted from kernels by a modified hot borate method [25] with a minor adaptation using 0.3 mg of ground tissue instead of the published 1 mg, and all volumes through the extraction process were reduced accordingly. The extracted RNA was purified using DNase digestion with RQ1 RNase-Free DNase (Promega, Madison, WI, USA, Cat. No. M6101) according to manufacturer’s instructions, followed by cleanup using RNeasy Plant Mini Kit (Qiagen, Hilden, Germany, Cat. No. 74903) as instructed in the product guide. The quality of the extracted RNA was measured using an RNA 6000 Nano Kit (Agilent Technologies, Santa Clara, CA, USA, Cat. No. 5067-1511), and the concentration was determined using a NanoDrop Spectrophotometer ND-1000 (Agilent Technologies, Santa Clara, CA, USA). The first-strand cDNA was synthesized using SuperScript II Reverse Transcriptase (Invitrogen, Carlsbad, CA, USA, Cat. No. 18064014) according to the manufacturer’s instructions for 1 µg of total RNA and a total reaction volume of 20 µL and later quantified using a NanoDrop Spectrophotometer ND-1000 (Agilent Technologies, Santa Clara, CA, USA).

### 4.3. Gene Selection and Primer Design

Six genes were used in this study to identify the most stable reference gene(s) for RT-qPCR analyses of maize kernels in response to wounding and *A. flavus* inoculation. The selected genes have been used as internal controls in previous plant studies because of their constitutive expression [26]. Using the reference genome sequence (B73 AGPv4) [27], available in the online database Gramene [28], sequences of the following genes were downloaded *Actin-1* (*ACT1*—*Zm00001d010159*), *β-tubulin2* (*β-Tub2*—*Zm00001d010275*), *Elongation factor 1-alpha* (*EFIα*—*Zm00001d037905*), *Eukaryotic initiation factor 4A-2* (*eIF4A2*—*Zm00001d016351*), *Glyceraldehyde-3-phosphate dehydrogenase* (*GAPDH*—*Zm00001d035156*), and *TATA-box-binding protein 2* (*TATA*—*Zm00001d019598*).

The coding and genomic sequences for each candidate gene were aligned using the Clustal W algorithm in the Lasergene Megalign software (DNASTAR, Madison, WI, USA) to identify the gene structure (exon and introns lengths) and ensure efficient primer design. The primer sequences were selected only on exonic sequences, and the online tool Primer3 [29,30] was used to help select sequences that met set parameters for optimal amplification. The selected primers were designed to amplify a region ranging between 110 to 300 bp (optimum 220 bp); the primer length had a minimum of 24 bp and a maximum of 35 bp (optimum 27 bp); GC content between 40–60%; and a 2 bp GC clamp (Table 1). Before ordering the primers, the selected primer sequences were submitted to a BLAST search against the reference B73 genome sequence RefGen_v4 (http://ensembl.gramene.org/Tools/Blast?db=core) to ensure the uniqueness of the product amplified by the selected primer pair.

To confirm that the selected primers would amplify the correct region, the respective pairs were used to prime amplification in 100 ng of the first-strand cDNA in an end-point PCR reaction. For each genotype, one sample (out of the nine) of synthesized first-strand cDNA was selected randomly and used for all primer testing. The master mix for this reaction was prepared according to the manufacturer’s instructions for the Ex Taq Hot Start Version (TaKaRa Bio Inc., Kusatsu, Japan, Cat. No. RR006A). The amplification was carried out with initial denaturation at 95 °C for 5 min, followed by 35 cycles of a denaturation at 94 °C for 30 s, annealing for 30 s at the primer pair’s average annealing temperature, and an extension of 45 s at 72 °C; after competition of the last cycle, a final extension was carried at 72 °C for 5 min. To check for the amplification of a single product, the PCR products were loaded into a 1.5% agarose-TAE gel stained with ethidium bromide and run at 70 V for 45 min at room temperature. Gel images were obtained using the AlphaImager HP system (Protein Simple, San Jose, CA, USA).

### 4.4. Quantitative Real-Time PCR Conditions

All samples used in this study were pooled (200 ug of each sample; 36 samples total) and diluted in a five-fold serial dilution to create a standard curve, which was amplified via RT-qPCR using the Applied Biosystems StepOnePlus Real-Time PCR System (Thermo Fisher Scientific, Waltham, MA, USA). The Applied Biosystems Power SYBR Green PCR Master Mix (Thermo Fisher Scientific, Whatman, MA, USA, Cat. No. 4367659) was used for qPCR following the manufacturer’s guidelines for a reduced final reaction volume of 10 µL. The reactions were amplified in the MicroAmp Optical 96-Well Reaction Plate (Thermo Fisher Scientific, Whatman, MA, USA, Cat. No. N8010560) sealed with MicroAmp Optical 8-Cap Strips (Thermo Fisher Scientific, Whatman, MA, USA, Cat. No. 4323032). Each reaction was run with initial denaturation at 95 °C for 10 min, followed by 40 cycles of denaturation at 95 °C for 15 s, annealing for 30 s at the primer pair’s average personalized annealing temperature, and an extension of 15 s at 72 °C; after completion of the last the cycle, a melt curve analysis was performed according to the machine’s preset Step and Hold protocol (95 °C for 15 s; 60 °C for 1 min; temperature ramp increment of 0.3 °C up to 95 °C).

The Cq values obtained from technical replicates run for each point of the curve were averaged and plotted against the logarithm of the pooled cDNA dilution factors to create a linear regression equation. The amplification efficiency [4] of each gene was then calculated using the slope from the linear regression equation with the following formula: % E = (10^[−1/slope]^ − 1) × 100.

### 4.5. Determination of Expression Stability of Reference Genes

The expression profile of the candidate genes was plotted using R, version 1.2.1335 [31]. To evaluate the stability of gene expression for each candidate reference gene, three software packages were used geNorm [13], NormFinder [14], and BestKeeper [15]. The geNorm add-in for Microsoft Excel was used to calculate the expression stability value (M) and the pairwise variation (V) for each gene. Since geNorm calculates M and V based on the relative expression levels (ΔCq), the raw Cq of each sample was averaged over technical triplicates only; biological replicates were treated as different samples. Cq values averaged over technical reps were then converted using the formula: 2^−(ΔCq)^, where ΔCq = (sample’s averaged Cq—minimum Cq for analysis set). To perform stability calculations with NormFinder, the R software version 3.5.132 was used with the code provided by the authors in the original publication. The data input used for NormFinder was the same as that used for geNorm. The add-in for Microsoft Excel was used to calculate the stable gene expression using BestKeeper. This last software utilizes raw Cq values; therefore, the averaged values of technical triplicates were used as individual input data points.

### 4.6. Validation of Reference Genes

To validate the stability of the selected reference genes, the three most stable genes, *ACT1*, *EFIα*, and *eIF4A2*, and the least stable, *GAPDH*, were used to normalize the expression profile of the maize gene of interest (GOI) *Zm00001d020612* under the different genotypes and treatments. The qRT-PCR methods used were the same as described above. The primers used to amplify the GOI and its characteristics are described in Supplementary Table S1. The obtained quantitation cycle (Cq) from the RT-qPCR results was used to calculate the relative gene expression of the selected candidates’ genes using the 2^−ΔΔCq^ method proposed by Livak and Schmittgen [4]. When samples were normalized to more than one reference gene, the geometric mean value was taken and used for the calculation of the ΔΔCq [13]. Statistical analysis using one-way ANOVA (analysis of variance) and post-hoc Tukey at a confidence interval of 95% were performed using R, version 1.2.1335 [31]. None of the statistical analyses were significant (*p* > 0.05).

## Figures and Tables

**Table 1:** Selected candidate reference genes, their Gramene accession number (Gene Acc. No.), primers pair sequences and characteristics, expected amplicon sizes, and amplification efficiency (E %). Gene abbreviations will be used herein to refer to each candidate reference gene.

Gene Abbrev.	Gene Acc. No.	Primer Sequence 5′ → 3′ (Forward/Reverse)	n-mer (bp)	TM (°C)	Size (bp)	E %	R^2^
*ACT1*	Zm00001d010159	GCCTATCGTATGTGACAATGGCACTGG	27	61.0	188	101	0.999
		CCAAGAGAGGCATCCTGACACTGAAGT	27				
*β-Tub2*	Zm00001d010275	AGGGTATCGATCTCTCATCATCTGAACTGAATCC	34	62.4	120	95	0.921
		CCATCAGGTTTTCAGGTTTGCCACTCGC	28				
*EFIα*	Zm00001d037905	GCGACCACTCCCAAGTATTCCAAGGCC	27	65.4	196	108	0.988
		GGTCCAACCCTGCTTGAGGCTCTTGACC	28				
*eIF4A2*	Zm00001d016351	AGAGGAATCGTCCCACTATGCAAGGGC	27	63.4	276	92	0.995
		GCCCTGCTAAGTGGAGCTCAGGTTCTA	27				
*GAPDH*	Zm00001d035156	TGATGTTTTGATGTCCTGAGGTGC	24	63.0	168	105	0.988
		CCCCTGGGGATGCTAAATCTACAACG	26				
*TATA*	Zm00001d019598	TGACCTAGGTGCACATGGTATGGCTGG	27	63.4	144	93	0.956
		CGTCTGACAAGCCCACAGTTTCGCTG	26				

**Table 2:** Quantification cycle (Cq) details for the candidate reference genes over biological replications and treatments. Genes are organized in the table by the most stable (top) to least stable according to the stability index (ΔP = 75th Percentile – 25th Percentile) (bottom).

	25th Percentile	75th Percentile	Mean	Median	Std. Dev.	% CV	ΔP
*ACT1*	21.165	22.120	21.792	21.603	0.762	3.526	0.955
*β-Tub2*	29.515	30.510	30.062	29.888	0.910	3.045	0.995
*eIF4A2*	21.917	23.104	22.669	22.432	1.383	6.165	1.187
*TATA*	29.405	30.682	29.884	29.759	0.831	2.793	1.277
*EFIα*	18.398	19.892	19.316	18.946	1.336	7.051	1.494
*GAPDH*	25.949	37.101	31.324	28.656	5.674	19.801	11.152

**Table 3:** Stability values of each candidate reference gene as calculated by geNorm, NormFinder, and BestKeeper algorithms. Genes are ranked in order of most stable (top) to least stable (bottom) by the expression stability value (M) for geNorm, stability level for NormFinder, and standard deviation (SD) for BestKeeper.

geNorm		NormFinder		BestKeeper	
Gene	M	Gene	Stability	Gene	SD
*β-Tub2*	0.860	*β-Tub2*	0.250	*ACT1*	0.580
*ACT1*	0.907	*EFIα*	0.460	*β-Tub2*	0.680
*EFIα*	0.953	*eIF4A2*	0.680	*TATA*	0.680
*eIF4A2*	REMOVED	*ACT1*	0.820	*eIF4A2*	0.960
*TATA*	REMOVED	*TATA*	1.500	*EFIα*	1.050
*GAPDH*	REMOVED	*GAPDH*	5.400	*GAPDH*	5.500

## Data Availability

The data presented in this study are available on request from the corresponding author. The data are not publicly available due to privacy policies.

## Supplementary Materials

The following are available online at https://www.mdpi.com/article/10.3390/toxins13060386/s1, Figure S1: Melt curve for the candidate reference genes, Figure S2: Standard curve to calculate amplification efficiency of candidate reference genes, Table S1: Primers characteristics for the gene of interest *Zm00001d020612* used to validate reference candidate genes.
