# Supplementary material for: Reference Gene Selection for RT-qPCR Analysis in Maize Kernels Inoculated with Aspergillus flavus
Source: Toxins (Basel). 2021 May 28;13(6):386. doi: 10.3390/toxins13060386 (PMC8229600; doi:10.3390/toxins13060386)
Supplement: Supplementary file 1 [file toxins-13-00386-s001.zip › toxins-1200341-supplementary.pdf]

# Supplementary Materials: Reference Gene Selection for RT-qPCR Analysis in Maize Kernels Inoculated with *Aspergillus flavus*

**Dafne Alves Oliveira, Juliet D. Tang and Marilyn L. Warburton**

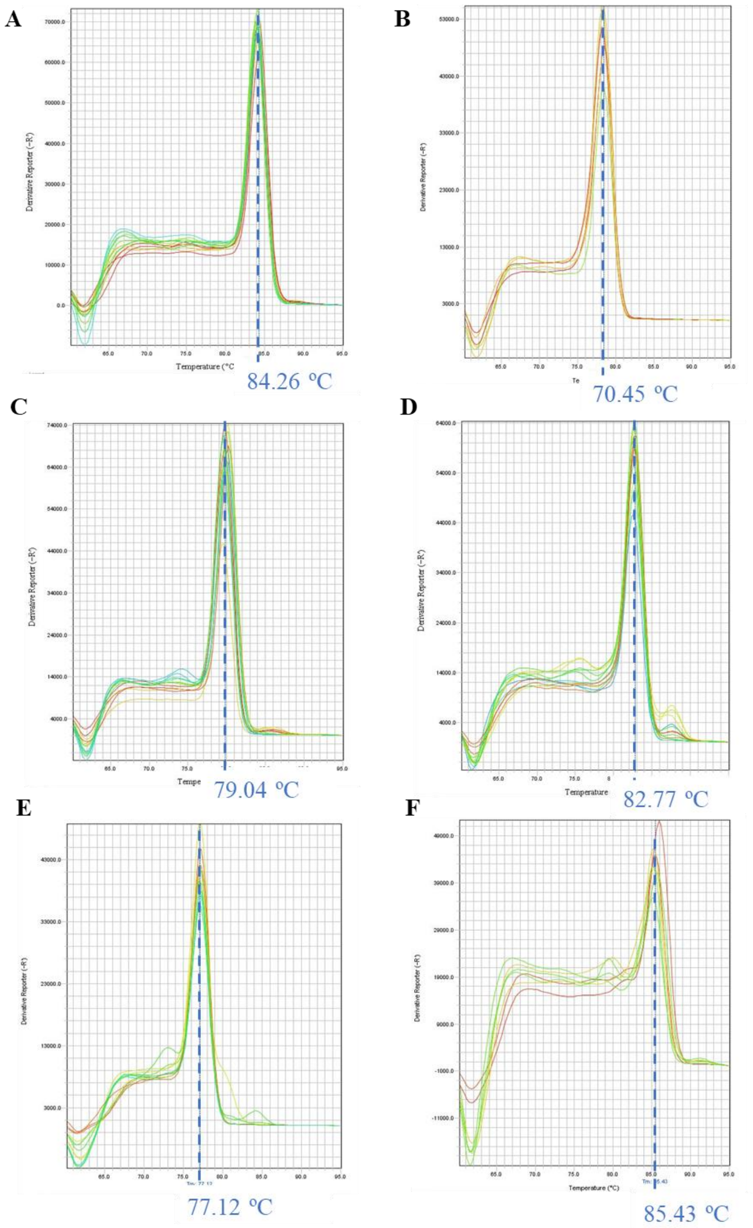


**Figure S1.** Melt curve for the candidate reference genes (**A**) *ACT1*, (**B**) *β-Tub2*, (**C**) *EFIα*, (**D**) *eIF4A2*, (**E**) *GAPDH* and (**F**) *TATA*.

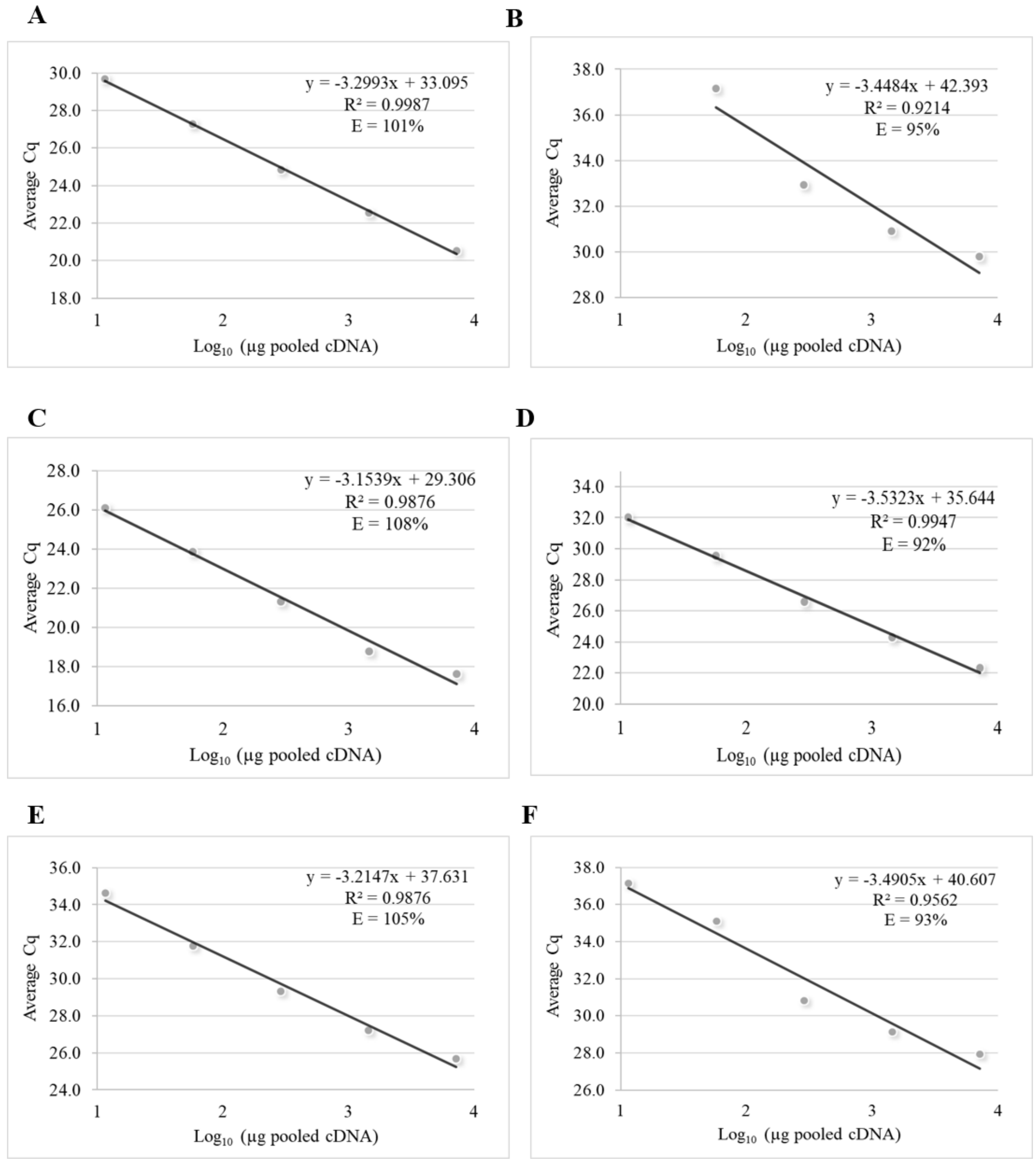


**Figure S2.** Standard curve to calculate amplification efficiency of candidate reference genes (**A**) *ACT1*, (**B**) *β-Tub2*, (**C**) *EFIα*, (**D**) *eIF4A2*, (**E**) *GAPDH* and (**F**) *TATA*.

**Table S1.** Primers characteristics for the gene of interest *Zm00001d020612* used to validate reference candidate genes.

| Gene Acc. No. | Primer Sequence 5' → 3' (Forward/Reverse) | n-mer (bp) | TM (°C) | Size (bp) |
|---|---|---|---|---|
| *Zm00001d020612* | GGTGACTTATGTTGCTCGTTTGAGACTGTTGC | 32 | 62.0 | 264 |
| | AGCAAAATGGGCCCGAAATATCAGAAGG | 28 | | |
